# Too Much Medicine

**Published:** 1884-09

**Authors:** K. P. Moore

**Affiliations:** Macon, Ga.


### TOO MUCH MEDICINE.

BY K. P. MOORE, M. D., MACON, GA.

No age, I suppose, in this world's history has been exempt from impositions of every form and variety. Every trade, calling and profession has been afflicted with superfluities of its peculiar kind. The legal profession has become burdened with laws and Supreme Court decisions so innumerable and multitudinous, that its members can find decisions to suit whatever phase or variety their cases may present.

Even in the sacred calling of the ministry, inventive and ingenious minds have so warped and distorted its legitimate construction, as to wring from the blessed volume doctrines to suit every fanciful caprice of man's fitful imagination. By way of parenthesis I would say, that the world looks anxiously to the *true* and *noble* in these two high and learned professions, for a faithful maintenance of the majesty and dignity of the laws, both human and divine.

But if ever a profession was afflicted with burdens more numerous and more grievous to be borne than those visited upon the Egyptians, it must be the medical profession. And while it may seem somewhat paradoxical for a profession, boasting of its progress and scientific attainments, to say that it is burdened with the very working tools with which it is endeavoring to shape its high destiny, yet it is certainly true, for all practical purposes at least, that our profession is burdened with too much medicine. We are overdosed, and are considerably debilitated from "too much *strong* medicine."

A rough estimate of the number of officinal medicines and appliances noticed in the last edition of the U. S. Dispensatory, numbers them over seventeen thousand. It is safe to say that *hundreds* of others find their way into the journals that are not mentioned in the dispensatory. There are over five thousand patent medicines, many of which are prescribed and recommended by so-called doctors. One is bewildered in contemplating this vast array of medicine. And when we remember the insatiable desire of the human family to be always taking something, and especially the readiness with which they run after "new drugs," we are left to wonder that the poor human stomach so long survives this eternal deluge of drugging and drenching.

Is it any wonder that a doctor never takes *any* medicine himself, nor gives his family any, when he has this picture before his eyes?

I am a strong advocate for enterprise, for investigation, and for chemical research; and I recognize the obligation of our profession to proper and legitimate chemical labor. But thousands of these so-called "new remedies" owe their existence to sensational introduction by enterprising and energetic manufacturers, from motives of a purely mercenary character, with but little if any regard to their real medicinal virtues, or to the relief which they may bring to suffering humanity. A great many of these remedies find here and there friends and admirers in our ranks, who, in consideration of a neat sample lot, and may be without sufficient trial, give favorable opinions of them. These favorable opinions are seized upon by the proprietors or manufacturers, and by a judicious and elaborate use of printers' ink, are forced into notice, and ultimately find their way into officinal recognition. I do not deny that an article thus introduced may occasionally have real merit, and may be worthy of professional confidence. But taken as a whole we may safely say,

> "Broad is the road that leads to death,
> And thousands walk together there,
> While wisdom shows a narrow path
> With *here* and *there* a traveler."

The object of this paper is not to antagonize progress. No profession has been more alive to progression and scientific investigation in every direction than ours, and none deserves more credit for attainments already achieved. But in our haste to *progress*, and in our eagerness to be "always on the front line," we are apt to become the character whom St. Paul credits with "having zeal without knowledge."

It is a mistake to measure a doctor by the number of new and unusual medicines which he incorporates into his prescriptions. At least it does not follow that he is the best balanced, or safest doctor, who makes the biggest effort to get the "latest" into his practice. A president of the American Medical Association recently said, that out of the hundreds of remedies given as improved diuretics, there were few, if any, equal to the old cream tartar, and yet said he, "the man who would now give cream tartar would be considered rather antiquated." The trouble is, converts to the many "new remedies" are too easily made. The older we get in the practice the less confidence we have in medicines, *per se*, and the *more* we have in *vis medicatrix naturæ.*

In the early morning of our professional lives, while pondering over our materia medica and therapeutics, and finding so many excellent remedies suggested for every ailment, we wonder at the ignorance which we feel must have kept the older doctors from curing *all* their patients. "But soon we learn 'twas all a dream."

Our youthful ardor falls below zero, and the implicit confidence with which we wrote *all* our prescriptions begins to pale before our sad disappointments, and we wake up to realize that many of the so-called *reliable* remedies have been weighed in the balance and found wanting.

The tradition, "long felt want," with the busy practitioner, is a few "true and tried" remedies, with whose virtues he is familiar, and whose effects he can confidently look for when prescribed.

A thousand new things may be introduced as cathartics and laxatives, and they may all be "purely vegetable," and, after all, perhaps none of them be superior, and few equal, to the *old* Epsom salts.

But by way of parenthesis, I would remark, that in writing your prescription you should write it Sulph. Magnesia, for if you write Epsom salts the whole family may remind you that *any old woman* could have given Epsom salts.

We are too prone to blindly accept what may be written about certain medicines as settled facts, without carefully watching their effects and determining for ourselves whether or not they possess the virtues accredited to them. In one or two instances our patient improves after taking certain remedies, the medicine gets the credit for the improvement, when in fact the patient may have gotten well quicker *without* the medicine. As before remarked, what we want is *less* drugs, *numerically*, and *more reliable* ones *practically*. For instance, if the indication in any given case is for a diuretic, we want *not more* than a half dozen good, thoroughly tested diuretics from which to make our selection. If we want a cardiac sedative we do not care to be burdened with forty or a hundred uncertain remedies which some energetic manufacturer offers as cardiac sedatives, with which to experiment upon our patients. And so on through the whole list of indications.

In hospitals and other places affording ample opportunity for experimentation, it might be well to thoroughly test some of these new remedies for which so much is claimed, and should one be found "worthy and well qualified." set it down as *one* of the *few* in which confidence may be placed in the treatment of whatever disease it may have proven itself meritorious. But let not the strange and euphonious names, or the enthusiastic effort of recent proprietors entice us into blindly accepting every new thing coming before us, without first following the injunction of the Divine lawgiver, and "prove all things."

